# Impact of tourniquet during knee arthroplasty: a bayesian network meta-analysis of peri-operative outcomes

**DOI:** 10.1007/s00402-020-03725-8

**Published:** 2021-01-08

**Authors:** Filippo Migliorini, Nicola Maffulli, Paolo Aretini, Andromahi Trivellas, Markus Tingart, Jörg Eschweiler, Alice Baroncini

**Affiliations:** 1 Department of Orthopaedic Surgery, RWTH Aachen University Clinic, Pauwelsstraße 30, 52074 Aachen, Germany; 2 Department of Medicine, Surgery and Dentistry, University of Salerno, Via S. Allende, 84081 Baronissi, SA Italy; 3 School of Pharmacy and Bioengineering, Keele University School of Medicine, Thornburrow Drive, Stoke on Trent, England; 4 Barts and the London School of Medicine and Dentistry, Centre for Sports and Exercise Medicine, Queen Mary University of London, Mile End Hospital, 275 Bancroft Road, London, E1 4DG England; 5 Fondazione Pisana per la Scienza, Via Ferruccio Giovannini, 13, 56017 San Giuliano Terme, Pisa, Italy; 6 Department of Orthopaedics, David Geffen School of Medicine at UCLA, 10833 Le Conte Ave, Los Angeles, CA 90095 USA

**Keywords:** Bayesian network meta-analysis, Knee arthroplasty, Tourniquet

## Abstract

**Introduction:**

The role of tourniquet during knee arthroplasty is controversial. The present study compares various tourniquet protocols using a Bayesian network meta-analysis of peri-operative data.

**Material and methods:**

The present study was conducted in accordance with the PRISMA extension statement for reporting systematic reviews incorporating network meta-analyses of health interventions. The literature search was conducted in September 2020. All clinical trials investigating the role of tourniquet in knee arthroplasty were considered for inclusion. Methodological quality was assessed using Review Manager 5.3. A Bayesian hierarchical random-effects model analysis was used in all comparisons.

**Results:**

Ultimately, pooled data from 68 studies (7413 procedures) were analysed. Significant inconsistency was found in the data relating to total estimated blood lost; no assumption could be made on this outcome. Full-time tourniquet resulted in the shortest surgical duration and lowest intra-operative blood lost, in both cases followed by incision-to-suture. The incision-to-suture protocol achieved the smallest drop in haemoglobin during the first 72 h post-operatively and the lowest rate of blood transfusion, both followed by full-time tourniquet. Hospitalisation was shortest in the absence (no-tourniquet) group, followed by the cementation-to-end group.

**Conclusion:**

For knee arthroplasty, longer tourniquet use is associated with the shorter duration of surgery, lower intra-operative blood lost, lower drops in haemoglobin and fewer transfusion units. The shortest average hospitalisation was associated with no tourniquet use.

## Introduction

The use of pneumatic tourniquet in surgery was first described by Sir Harvey Cushing in 1904. [1] Tourniquets are used frequently in orthopaedic procedures. The American Association of Hip and Knee Surgeons has reported that approximately 95% of surgeons use tourniquets in some form during knee arthroplasty [2]. Using a tourniquet reduces intraoperative blood loss [3, 4] and optimizes visualisation, thereby shortening surgical duration [5, 6]. It has been also hypothesized that the use of tourniquet may improve cement penetration, but results from clinical trials are controversial [7–9]. Between no tourniquet and full-time use of tourniquet, various timing protocols are routinely advocated: from skin incision to cement hardening, only during the cementation phase, from cement hardening to the end of procedure, from incision to wound closure. Despite the high number of clinical trials and reviews addressing tourniquet use, no consensus has been reached regarding the optimal tourniquet protocol for knee arthroplasty [10–14]. To date, no studies have been performed comparing peri-operative outcomes associated with the most common tourniquet protocols during knee arthroplasty. The present Bayesian network meta-analysis was therefore conducted to study the following outcomes: duration of surgery, intraoperative blood loss, total estimated blood loss, haemoglobin drop, blood units transfused, length of hospitalisation.

## Material and methods

### Search strategy

The present Bayesian network meta-analysis was conducted in accordance with the PRISMA extension statement for reporting systematic reviews incorporating network meta-analyses of health interventions [15]. The PICO algorithm was defined as.

P (Population): primary knee arthroplasty;

I (Intervention): effect of tourniquet;

C (Comparison): tourniquet duration;

O (Outcomes): peri-operative data.

### Literature search

The literature search was performed independently by two authors (FM, AB). In September 2020, the main online databases were accessed: Pubmed, Google Scholar, Scopus, EMBASE. The following keywords were used in combination: *knee, arthroplasty, replacement, prosthesis, outcomes, treatment, surgery, therapy, tourniquet, surgical, hospitalisation, length duration, transfusion, blood lost, hb, haemoglobin.* Resulting titles were screened and their abstracts read. If the study was of interest, the full text was accessed. Bibliographies were also screened. Disagreements between reviewers were solved by a third author (MT).

### Eligibility criteria

All clinical trials evaluating the role of tourniquets during knee arthroplasty were considered. Following the Oxford Centre for Evidenced-Based Medicine (OEBM) [16], only articles with level I to III evidence were included for analysis. The search was limited to articles in English, Italian, German, French and Spanish, according to the language capabilities of the authors. Various types of implant were considered (e.g., cemented or uncemented, uni-compartimental or total component, cruciate retaining or bi-retaining or sacrificing), as were all common surgical approaches (e.g. medial parapatellar, quadriceps sparing, mid-vastus, sub-vastus). No distinction was made between minimally invasive and standard surgery. Different types of tourniquets and inflation pressures, as well as interventions incorporating navigation systems, were also considered. Reviews, case series, editorials, letters and expert opinions were not considered, nor were biomechanical, animal and cadaveric studies. Also excluded were data from knee arthroplasties following any kind of trauma, revision surgeries, and articles missing quantitative data concerning the outcomes of interest.

### Outcomes of interest

Two independent authors (FM, AB) extracted the data of interest. The following demographics were collected: author, year, journal, type of study, type of implant, tourniquet protocol, tourniquet pressure, number of procedures, mean age and BMI, percentage of female gender. Outcomes of interest were hospitalisation length, duration of surgery, intraoperative and total estimated blood loss, haemoglobin (Hb) values, transfusion rate of packed red blood cells.

### Methodological quality assessment

The methodological quality assessment was performed using Review Manager 5.3 (Nordic Cochrane Collaboration, Copenhagen) and its risk-of-bias summary tool. Six items from each study were evaluated: randomization (selection bias), allocation (selection bias), blinding method (detection bias), selective reporting (attrition bias), incomplete data (reporting bias), and unknown source of bias.

### Statistical analysis

Statistical analyses were performed by the main author (FM). To evaluate the demographic baseline of the included studies an analysis of variance (ANOVA) was performed using IBM SPSS Software, with values of *P* > 0.5 considered satisfactory. For the Bayesian network meta-analysis of comparisons and related effect, we used STATA Software/MP 14.1 (2015. Stata Corporation, College Station, TX: Stata LP). Network comparisons were performed using a hierarchical random-effects model analysis. Dichotomic variables were analysed through the log odds-ratio (LOR) method and continuous variables through the inverse variance method with a standardised mean difference (SMD) effect measure. Inconsistency was measured using the equation for global linearity via the Wald test. If *P* value ≥ 0.5, the null hypothesis could not be rejected and the consistency assumption could be accepted at the overall level of each treatment. The confidence interval (CI) and percentile interval (PrI) were set at 95%. An edge plot was drawn for each comparison to display both direct and indirect comparisons, contribution weights and network connections. The final effect of each treatment was displayed in the interval plot, while funnel plots were performed for each comparison to evaluate the risk of publication bias.

## Results

### Search result

**Fig. 1:**
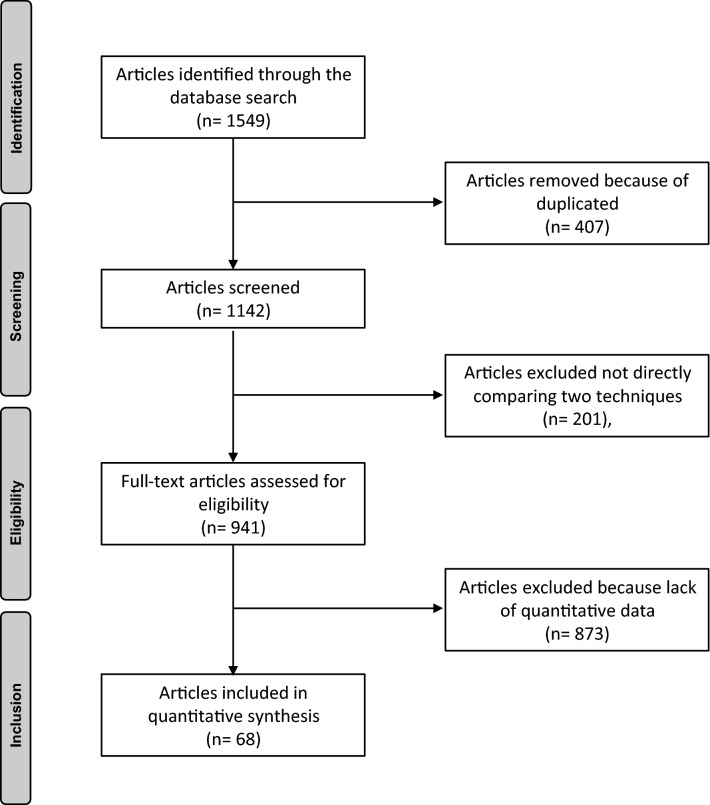
Flow-chart of the literature search

The initial search resulted in 1549 articles. 407 were duplicates. A further 1074 studies were excluded because of no direct comparison (*N* = 201), language limitations (*N* = 47), level of evidence (*N* = 388), type of study (*N* = 185), revision setting (*N* = 53), traumatology (*N* = 61), lack of quantitative data under the outcomes of interest (*N* = 113), uncertain data (*N* = 19) or other (*N *= 7). Finally, 68 studies were included: 45 randomized clinical trials, 9 prospective and 14 retrospective cohort studies. The flowchart of the literature search is shown in Fig. 1.

### Methodological quality assessment

**Fig. 2:**
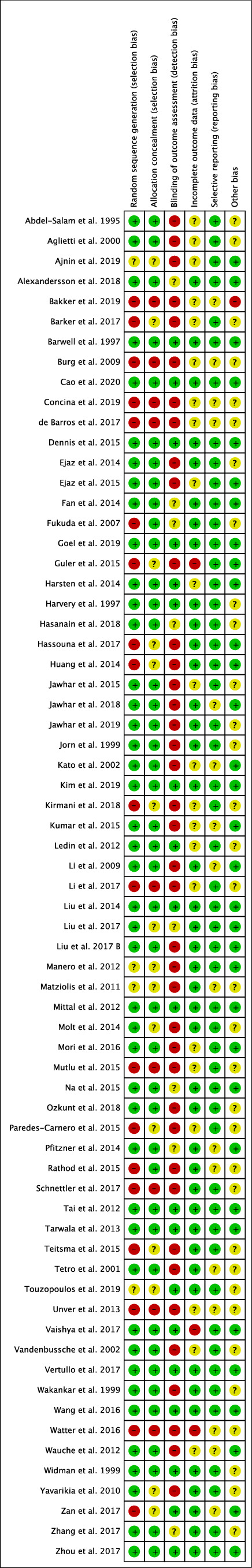
Cochrane risk of bias summary

The Cochrane risk of bias summary evidenced some important strengths and limitations of our meta-analysis. Approximately 65% of the included articles provided randomization, the most strength in the present study. The main limitation was the reduced number of studies using a blinding method. Risk of detection, attrition and reporting biases were all low. The overall methodological quality score was very good. The Cochrane risk of bias summary is shown in Fig. 2.

### Patient demographics

In total, data from 7413 procedures were analysed including 2330 procedures where no tourniquet was used. The mean age in this group was 67.87 ± 3.5 years, mean BMI was 28.89 ± 2.6 kg/m^2^, and 64% (*N* = 2330) were females. In the incision-to-cementation group, 953 procedures were analysed. The mean age was 68.27 ± 3.3 years, mean BMI 29.05 ± 2.9 kg/m^2^; 71% (*N* = 679) were females. In the cementation-to-end group, 782 procedures were analysed. The mean age was 66.94 ± 3.3 years, mean BMI 29.16 ± 2.3 kg/m^2^; 68% (*N* = 782) were females. In the incision-to-wound closure group, 455 procedure were analysed. The mean age was 71.21 ± 5.1 years, mean BMI 29.09 ± 1.0 kg/m^2^; 64% (*N* = 292) were females. In the full-time tourniquet group 2695 procedure were analysed. The mean age was 68.24 ± 2.9 years, the mean BMI 28.62 ± 2.4 kg/m^2^; 66% (*N* = 1775) were females. The ANOVA test detected optimal baseline comparability between the patient demographics according to age (*P* = 0.7), BMI (*P* = 0.8) and gender (*P* = 0.6). Demographics are shown in Table 1.

**Table 1:** Generalities and demographics of included studies

Author, year	Journal	Type of study	Type of implant		Tourniquet protocol	Tourniquet pressure (mmHg)	Samples (n)	Mean Age (years)	Female (%)	BMI (g/m^*2*^)
Abdel-Salam et al. [17]	J Bone Joint Surg	RCT	PS	Cemented	Absence		40	72.00	57.50	
					Full-time	SBP doubled	40	74.00	62.50	
Aglietti et al. [18]	Cin Orthop Rel Res	RCT		Cemented	Incision to cementation		10	70.00	70.00	
					Absence		10	68.00	60.00	
Ajnin et al. [19]	J Clin Orthop Trauma	PCS	CR	Cemented	Absence		29	73.00		33.00
					Full-time	300	29	73.00		33.00
Alexandersson et al. [20]	Knee Surg Sports Traumatol Arthrosc	RCT	Mixed	Cemented	Absence		43	69.70	48.83	27.90
					Full-time	300	38	68.00	52.63	28.60
Ayik et al. [21]	*J Knee Surg*	RCT	PS	Cemented	Absence		33	64.90	57.57	30.31
					Full-time	SBP + 100	32	65.39	56.25	31.38
Bakker et al. [22]	Turk J Anaesthesiol Reanim	RCS			Full-time	SBP + 50	300	65.00	64.33	28.90
					Absence		300	65.00	58.33	29.20
Barker et al. [23]	J Orthop Traumatol	RCS		Uncemented	Absence		53	62.00	52.83	33.20
					Incision to cementation	300	51	63.00	64.70	34.40
de Barros et al. [24]	Rev Bras Ortop	RCS	CR	Cemented	Full-time		75	67.00	73.30	
					Absence		42	67.00	71.43	
Barwell et al. [25]	J Bone Joint Surg	RCT	Mixed	Cemented	Full-time		44	71.00	65.90	
					Incision to cementation		44	69.00	72.72	
Burg et al. [26]	J Musc Res	RCS	CR	Cemented	Full-time		49	71.60	79.22	
					Cementation to end		28	72.50	79.22	
Cao et al. [27]	J Orthop Surg	RCT	PS	Cemented	Full-time		51	64.90	72.54	24.36
					Cementation to end		51	65.20	74.51	24.52
Concina et al. [28]	Acta Biomed	RCS	PS	Cemented	Incision to wound suture		51	73.00	61.76	
					Cementation		50	70.00	68.00	
Dennis et al. [29]	Clin Orthop Relat Res	RCT			Full-time	250	28	62.00	42.85	29.00
					Absence or cementation	250	28	62.00	42.85	29.00
Ejaz et al. [30]	Acta Orthop	RCT	CR	Cemented	Absence		31	68.00	45.16	25.00
					Full-time	250	33	68.00	45.45	25.00
Ejaz et al. [31]	Arthroplasty J	RCT	CR	Cemented	Absence		28	68.20	46.43	25.20
					Full-time	250	29	68.30	55.17	25.10
Fan et al. [32]	Knee	RCT	PS	Cemented	Full-time	SBP + 100	30	65.37	76.66	27.24
					Cementation	SBP + 100	30	63.27	70.00	26.26
Fukuda et al. [33]	Arch Orthop Trauma Surg	PCS		Cemented	Absence		21	73.10	85.71	26.50
					Full-time	350	27	71.20	85.19	26.10
Goel et al. [34]	J Bone Joint Surg	RCT	CR	Cemented	Absence		100	65.50	52.00	31.30
					Full-time	225–300	100	66.00	50.00	30.90
Guler et al. [35]	Knee Surg Sports Traumatol Arthrosc	RCS	PS	Cemented	Full-time		70	67.40	85.71	
					Absence		78	65.80	82.05	
Harsten et al. [36]	Knee	RCT	CR	Cemented	Absence		32	66.00	43.75	28.39
					Full-time		32	68.00	46.87	27.41
Harvery et al. [37]	Arthroplasty J	RCT		Cemented	Absence		28	73.40		
					Cementation		16	72.40		
					Full-time		36	68.30		
Hasanain et al. [38]	Arthroplasty J	RCT	PS	Cemented	Full-time		54	62.93	63.00	32.41
					Cementation	SBP + 100–150	54	62.93	63.00	32.41
Hassouna et al. [39]	EC Orthop	PCS	CR	Cemented	Full-time	300	48	69.00	66.66	29.30
					Cementation to end		104	69.00	55.33	28.80
Huang et al. [40]	Arch Orthop Trauma Surg	PCS		Cemented	Full-time	SBP + 100	30	66.20	66.66	26.10
					Incision to cementation		30	66.10	66.66	25.90
					Cementation		30	66.30	63.33	26.50
Jawhar et al. [41]	Knee Surg Sports Traumatol Arthrosc	RCT		Cemented	Full-time	380	15	70.60	53.33	32.10
					Absence		15	70.60	53.33	33.80
Jawhar et al. [42]	Knee Surg Sports Traumatol Arthrosc	RCT		Cemented	Full-time	360	43	70.00	62.79	31.90
					Absence		43	71.00	62.79	31.90
Jawhar et al. [43]	Knee Surg Sports Traumatol Arthrosc	RCT		Cemented	Full-time	360	50	69.30	34.00	31.90
					Absence		49	68.30	61.22	31.40
Jorn et al. [44]	Acta Orthop Scand	RCT	PS	Mixed	Full-time	300	35	71.00	57.14	27.96
					Incision to wound suture		42	71.00	78.57	28.78
Kato et al. [45]	Anesthesiology	RCT			Absence		24	63.00		
					Full-time	350	22	65.00		
Kim et al. [46]	BMC Musc Dis	RCT	mixed	Cemented	Incision to cementation	255	80	71.00	91.00	27.10
					Incision to cementation	233.9	80	71.80	85.00	27.40
Kirmani et al. [47]	Int J Res Orthop	PCS		Cemented	Full-time		52	69.80	67.30	
					Absence		146	73.00	65.80	
Kumar et al. [48]	J Clin Orthop Trauma	RCT			Incision to wound suture	SBP + 100	30	58.00	70.00	
					Absence		30	58.00	70.00	
Ledin et al. [49]	Acta Orthop	RCT	CR	Cemented	Full-time	275	25	70.00		29.00
					Absence		23	71.00		28.00
Li et al. [50]	Int Orthop	RCT	PS	Cemented	Full-time	SBP + 100	40	71.00		27.30
					Absence		40	70.00		26.80
Li et al. [51]	Medicine	RCS			Full-time		94	65.34	90.40	24.50
					Absence		36	65.08	83.30	25.00
Liu et al. [52]	Knee Surg Relat Res	RCT	PS	Cemented	Full-time	300	10	67.00	30.00	25.57
					Absence		10	70.00	10.00	27.09
Liu et al. [53]	Int J Clin Exp Med	RCT	PS	Cemented	Full-time	SBP + 125	26	65.80	69.23	28.20
					Absence		26	65.80	69.23	28.20
Liu et al. [54]	Orthop Surg	RCT	PS	Cemented	Full-time	SBP + 125	52	67.00		28.10
					Absence		52	67.00		28.10
Manero et al. [55]	Rev Esp Anestesiol Reanim	PCS		Cemented	Incision to wound suture	280	48	72.69	62.50	
					Full-time		48	71.54	70.80	
Matziolis et al. [56]	Orthopäde	RCS		Cemented	Absence	300	285	67.70	58.24	
					Full-time		262	68.50	59.92	
Mittal et al. [57]	J Surg	RCT		Cemented	Cementation	300	31	67.50	81.00	32.50
					Incision to cementation	300	34	66.60	74.00	32.60
Molt et al. [58]	Knee	RCT	Mixed	Cemented	Full-time	300	30	70.00	53.30	28.00
					Absence		30	67.00	53.30	28.00
Mori et al. [59]	Knee	RCT	PS	Cemented	Full-time	250	51	72.80	88.23	27.70
					Absence		52	74.60	82.69	29.20
Mutlu et al. [60]	Int J Surg	RCS	PS	Cemented	Incision to cementation	SBP + 150	61	67.20	78.10	
					Absence		65	65.80	72.20	
Na et al. [61]	Knee Surg Sports Traumatol Arthrosc	RCT	PS	Cemented	Incision to cementation	253	105	72.00	94.00	26.80
					Short deflation before capsule closure	256	101	73.00	93.00	26.90
Ozkunt et al. [8]	Medicine	RCT	CR	Cemented	Full-time		24	65.05	100.00	
					Cementation		20	65.05	100.00	
					Absence		25	65.05	100.00	
Paredes-Carnero et al. [62]	Rev Esp Cir Ortop Traumatol	RCS	PS	Cemented	Incision to wound suture	250–370	101	73.52	30.00	
					Full-time	250–370	100	75.62	30.00	
Pfitzner et al. [63]	Knee Surg Sports Traumatol Arthrosc	RCT	PS	Cemented	Full-time	350	45	69.30	53.33	27.80
					Absence		45	70.50	75.55	26.00
Rathod et al. [64]	J Knee Surg	PCS	PS	Cemented	Full-time		40	64.10	57.50	31.60
					Cementation		40	63.60	50.00	29.10
Schnettler et al. [65]	J Bone Joint Surg	RCS		Cemented	Cementation	250	45	64.69	67.00	30.24
					Absence		36	65.60	69.00	31.39
Tai et al. [66]	J Bone Joint Surg	RCT		Cemented	Incision to wound suture	SBP + 100	36	72.10	75.00	28.60
					Absence		36	72.50	77.77	27.90
Tarwala et al. [67]	Clin Orthop Relat Res	RCT	PS	Cemented	Incision to cementation	250	39	66.10	56.41	29.90
					Cementation	250	40	64.60	55.00	31.40
Teitsma et al. [68]	Orthop Muscular Syst	PCS	PS	Cemented	Absence		47	65.00	51.06	27.10
					Full-time	250	49	63.00	67.34	28.39
Tetro et al. [69]	Canadian J Surg	RCT			Incision to cementation	SBP + 125–150	33	69.80	54.54	
					Absence		30	69.80	63.33	
Touzopoulos et al. [70]	Eur J Orthop Surg Traumatol	RCS	CR	Cemented	Absence		50	69.92	84.00	31.32
					Full-time	350	50	70.73	84.00	31.04
Unver et al. [71]	Orthop Nur	PCS	CR	Cemented	Full-time	AOP	17	68.00	82.35	30.80
					Full-time	300	21	67.30	85.71	32.00
Vaishya et al. [72]	J Clin Orthop Trauma	RCT		Cemented	Incision to cementation	SBP + 150	40			
					Cementation	SBP + 150	40			
Vandenbussche et al. [73]	Int Orthop	RCT	PS	Cemented	Full-time	350	40	72.50	77.50	
					Absence		40	68.50	60.00	
Vertullo et al. [7]	J Orthop Surg	RCT	PS	Cemented	Cementation	300	20	67.85	50.00	30.43
					Absence		20	65.65	45.00	31.00
Wakankar et al. [74]	J Bone Joint Surg	RCT		Cemented	Full-time	SBP doubled	37	72.50	70.27	
					Absence		40	71.80	65.00	
Wang et al. [75]	Knee Surg Sports Traumatol Arthrosc	RCT		Cemented	Incision to cementation	SBP + 100	25	72.30	80.00	28.80
					Cementation	SBP + 100	25	72.50	84.00	29.10
Watter et al. [76]	Reconstr Rev	RCS		Cemented	Absence		100	63.80	57.00	29.80
					Cementation		100	67.30	65.00	28.40
					Incision to cementation		100	63.00	63.00	31.90
Wauche et al. [77]	Arch Orthop Trauma Surg	RCT		Uncemented	Full-time	SBP + 100	19	63.20		
					Absence		18	61.40		
Widman et al. [78]	Acta Orthop Scand	RCT	CR	Cemented	Incision to wound suture	300–350	46	72.00	76.08	
					Full-time	300–350	39	71.00	71.79	
Yavarikia et al. [79]	Pak J Biol Sci	RCT	CR	Cemented	Absence	220–275	31	66.00	75.86	
					Incision to cementation	220–275	36	64.00	72.72	
Zan et al. [80]	Bone Joint Res	RCS	PS	Cemented	Full-time	220–275	29	68.00	72.72	
					Incision to cementation	250	196	69.40	57.14	25.70
					Full-time	250	200	69.60	60.50	26.10
Zhang et al. [81]	Ir J Med Sci	RCT		Cemented	Full-time	317	50	70.30	52.00	29.30
					Incision to cementation	316	50	71.00	60.00	29.10
					Osteotomy to end	322	30	68.20	83.33	29.60
Zhou et al. [82]	J Orthop Surg Res	RCT	PS	Cemented	Absence		68	69.10	89.71	25.70
					Full-time		72	66.80	81.94	26.10

*SBP* systolic blood pressure, *AOP* arterial occlusion pressure

### Outcomes of interest

**Fig. 3:**
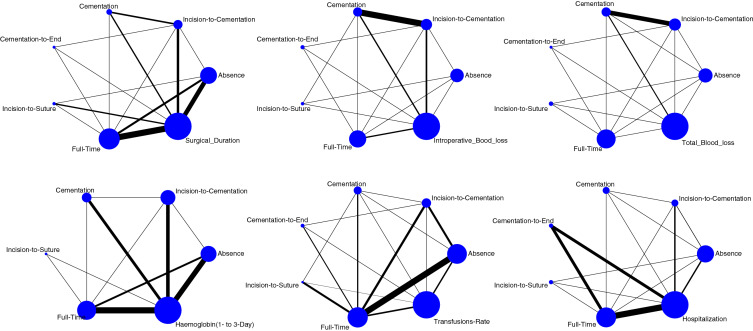
Edge plots of the comparisons

**Fig. 4:**
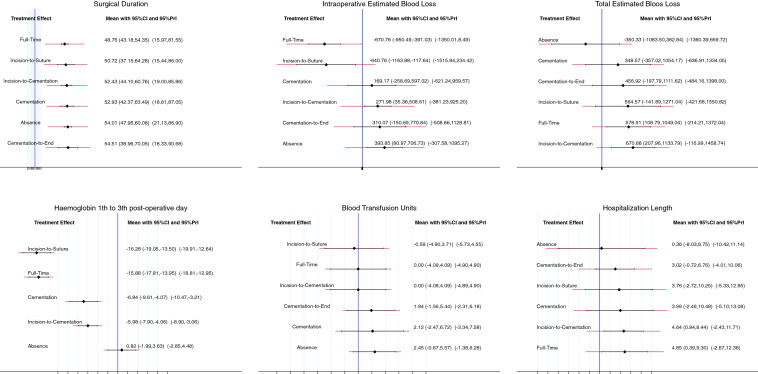
Interval plots of comparisons

**Fig. 5:**
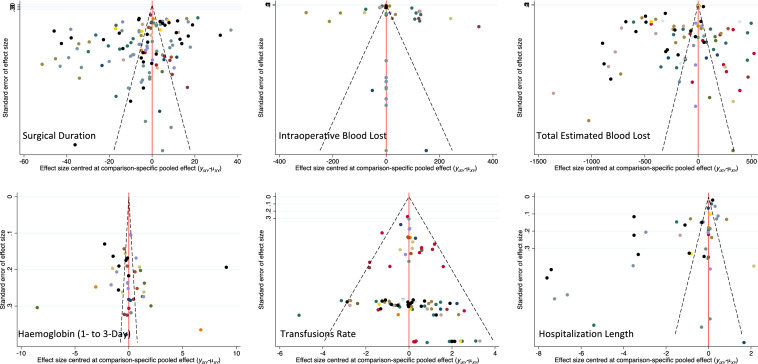
Funnel plots of the comparisons

Edge, interval and funnel plots are shown in Figs. 3, 4, 5, respectively. The shortest average duration of surgery was observed in the full-time tourniquet group (SMD: 48.76; 95% CI 43.18–54.35), followed by the incision-to-suture group (SMD: 50.72; 95% CI 37.15–64.28). The cementation-to-end group showed the longest operation time (SMD: 54.51; 95% CI 38.96–70.05), followed by the absence-of-tourniquet group (SMD: 54.01; 95% CI 47.95–60.08). The test for overall inconsistency was not significant (*P* = 0.7).

Intraoperative blood lost was lowest in the full-time tourniquet group (SMD: -670.76; 95% CI  – 950.49 to  – 391.03), followed by the incision-to-suture group (SMD:  – 640.76; 95% CI  – 1163.88 to  – 117.64). The absence of tourniquet group reported the highest intraoperative blood loss (SMD: 393.85; 95% CI 80.97–706.73), followed by the cementation-to-end group (SMD: 310.07; 95% CI  – 150.69 to 770.84). The test for overall inconsistency was not significant (*P* = 0.8).

The absence of tourniquet group showed the lowest amount of total estimated blood lost (SMD:  – 350.33; 95% CI  – 1083.50 to 382.84), followed by the cementation only group (SMD: 348.57; 95% CI  – 357.02 to 1054.17). The incision-to-cementation group had the highest amount of total estimated blood loss (SMD: 670.88; 95% CI 207.96–1133.79), followed by the full-time group (SMD: 578.91; 95% CI 108.79–1049.04). The test for overall inconsistency was significant and the consistency assumption could not be accepted (*P* = 0.009).

The incision-to-suture group showed the lowest haemoglobin drop 72 h after surgery (SMD:  – 16.28; 95% CI  – 19.05 to  – 13.50), followed by the full-time group (SMD:  – 15.88; 95% CI  – 17.81 to  – 13.95). The absence group of tourniquet showed the highest haemoglobin drop 72 h after surgery (0.82; 95% CI  – 1.99 to 3.63), followed by the incision-to-cementation group (SMD:  – 5.98; 95% CI  – 7.90 to  – 4.06). The test for overall inconsistency was not significant (*P* = 0.7).

The transfusion rate of packed red blood cells was lowest in the incision-to-suture group (LOR:  – 0.59; 95% CI  – 4.90 to 3.71), followed by the full-time group (LOR: 0.00; 95% CI  – 4.09 to 4.09). The absence of tourniquet group showed the highest rate of blood transfusion (LOR: 2.45; 95% CI  – 0.67 to 5.57), followed by the cementation only group (LOR: 2.12; 95% CI  – 2.47 to 6.72). The test for overall inconsistency was not significant (*P* = 0.6).

The length of hospital stay was shortest in the absence of tourniquet group (SMD: 0.36; 95% CI  – 8.03 to 8.75), followed by the cementation-to-end group (SMD: 3.02; 95% CI  – 0.72 to 6.76). The longest hospitalisation length was reported for the full-time tourniquet group (SMD: 4.85; 95% CI 0.39–9.30), followed by the incision-to-cementation group (SMD: 4.64; 95% CI 0.84–8.44). The test for overall inconsistency was not significant (*P* = 0.5). 

## Discussion

According to the main findings of the present Bayesian network meta-analysis, longer tourniquet associated with shorter surgery duration, lower intra-operative blood lost, higher post-operative haemoglobin values, and lower rates of blood transfusion units following knee arthroplasty. A shorter average hospitalisation was found in the absence of tourniquet group. Regarding the endpoint total estimated blood loss, statistically significant inconsistency was found, and no assumption could be drawn.

The shorter surgery duration found in the full-time tourniquet group, followed by the incision-to-suture group was expected. Impaired visualization of anatomical structures and the need to constantly wipe the surgical field can lead to longer operating times. In a recent meta-analysis, Cai et al. [83] investigated the difference in surgical duration between absence of tourniquet and full-time tourniquet protocols, finding statistically significant shorter durations in the full-time group across 11 studies. Similar results were reported by Zhang et al. [3] in 2017 from eight studies comparing incision-to-suture versus a full-time tourniquet regime. Likewise, in 2019, Liu et al. [84] analysed operating times across several tourniquet protocols, evidencing significantly longer surgeries in the absence group compared to the full-time tourniquet group (*P* = 0.005). On the other hand, Wang et al. [12] found no difference in surgical duration between shorter tourniquet use during cementation only and longer tourniquet use across 338 procedures; however, their findings were compromised by a high level of heterogeneity across the data. Tie et al. [11] also found no significant difference between early versus late tourniquet release in over 930 procedures, but their findings too were compromised by a high level of heterogeneity.

Intra-operative blood loss was significantly less with longer tourniquet protocols. Since the purpose of the tourniquet is to limit intra-operative blood loss, these results are expected, and previous meta-analyses observed similar results. In 2019, Cai et al. [83] analysed intra-operative blood loss in a meta-analysis of 234 procedures, and found significantly less bleeding with full-time tourniquet use compared to the absence group. In 2018, Wang et al. [12] performed a meta-analysis comparing cementation only versus longer tourniquet use, and found significantly less intraoperative blood loss with long-term tourniquet use. Intraoperative blood loss was also analysed by Liu et al., in 2019, [84], who found that full-time tourniquet use was correlated with lower intraoperative blood loss compared to absence (1011 samples, *P* < 0.0001) and cementation only groups (323 samples, *P* < 0.0001). Previous meta-analyses have reported similar findings [85, 86].

Regarding the endpoint of total estimated blood lost, the equation for global linearity detected statistically significant inconsistency. Therefore, the assumption must be refused at the overall level of each treatment. This result must be interpreted in light of the limitations of the present study. Current evidences concerning total estimated blood lost are controversial. In 2019, Cai et al. [83] performed a meta-analysis of full-time tourniquet use compared to its absence. Across 98 patients, no differences found and a high level of heterogeneity was detected. Zhang et al. [3] found lower values of total blood loss with incision-to-suture tourniquet use compared to the full-time group. Wang et al. [12] found statistically significant lower total blood loss with long-term tourniquet use compared to the cementation group. Liu et al. [84] found no statistically significant differences concerning total estimated blood loss by comparing absence versus full-time and incision-to-suture versus full-time tourniquet use. Tie et al. [11] found lower blood loss in the late tourniquet release compared to early release across approximately thousand patients; this result was statistically significant, but a high level of heterogeneity was also detected.

In the present network analysis, haemoglobin values remained higher in the longer tourniquet procedures. This result can be explained by a lower amount of intra-operative blood loss. Recent meta-analyses have shown no differences among different protocols of tourniquet use during knee arthroplasty. Huang et al. [87] analysed full-time versus incision-to-suture protocols and found no difference in Hb level and Hb drop across 511 procedures. Haemoglobin drop was also evaluated in a recent meta-analysis of Tie et al. [11] over 518 procedures, with no differences between early and late tourniquet release.

In patients with Hb values under 6 g/dL, a blood transfusion is almost always required [88–90]. In patients with Hb values between 6 and 10 g/dL, with concomitant presence of symptoms indicative of hypoxia (e.g., tachycardia, hypotension, fatigue, sleepiness, dizziness), a transfusion may be required [88–90]. Patients with values over 10 g/dL very rarely need transfusion [88–90]. Transfusion of blood units was more frequent in the shorter tourniquet procedures compared to the longer ones. This analysis showed heterogeneity across studies; however, the overall results are consistent and reliable. Results from previous meta-analyses were not statistically significant. However, the blood transfusion rate and consumption of blood units were higher in the short tourniquet procedures than the longer ones. In 2019, Cai et al. [83] performed a meta-analysis comparing absence versus full-time tourniquet over eleven RCTs. They found any statistically significant difference in the rate of blood transfusion. Wang et al. [12] found no difference between the cementation and long-term tourniquet (*P* = 1) across 167 procedures. The 2019 meta-analysis by Liu et al. [84] found no statistical difference concerning transfusion rates. However, shorter tourniquet time resulted in a higher rate of transfusion (23.5%) compared with incision-to-suture (17.7%) and full-time tourniquet (20.4%). In 2017, Zhang et al. [3] compared incision-to-suture versus full-time tourniquet over 1010 procedures and found an increased rate of transfusion in the full-time group (13.4% versus 7.42%); however, these results were not statistically significant. Huang et al. [87] found no statistically significant difference concerning the transfusion rate between full-time (18.4% versus 22.1%) and incision-to-suture in over 256 patients.

Length of hospital stay was shorter in the absence of tourniquet group. This endpoint involved a large number of studies and was characterized by high heterogeneity with the equation for global linearity yielding a narrow result. The ranking showed a mix of short and long tourniquet protocols with wide CI and Prl; thus, the real effect of this endpoint must be considered with caution. The endpoint hospitalisation length has been included in only a few meta-analyses. The latest meta-analysis, performed by Huang et al. [87] in 2015, found no difference between incision-to-suture versus full-time tourniquet. Since shorter tourniquet time lead to less damage to the quadriceps, the post-operative function and pain without the use of a tourniquet may well be improved, consequently shortening the hospitalization length. However, this endpoint requires further investigations.

The present Bayesian network meta-analysis has several limitations and strengths. The analyses were performed with no regard for antifibrinolytic drugs (e.g., tranexamic acid) and thromboembolic prophylaxes (e.g., unfractionated heparin, oral anticoagulants). Furthermore, the utilisation of drains was not considered in the analyses. The use of tranexamic acid and the avoidance of drainages has been supposed to eradicate the use of tourniquet. However, this is still controversial [91, 92]. The effects of different tourniquet applications on cement penetration and implant anchorage were not evaluated, nor were type of prosthesis, technique, surgical approach, or length of skin incision. These limitations arise mostly from the lack of data in the literature. Therefore, no comprehensive analyses could be performed. Moreover, there was heterogeneity across articles concerning exclusion and inclusion criteria. For example, some differences in type of tourniquet and tourniquet pressure were detected. However, given the insufficient data, a separate analysis could not be performed. In conclusion, considering these limitations, the data from the present Bayesian network meta-analysis must be interpreted with caution. Points of strength of the present work are represented by the comprehensive nature of the literature search, the rigor of our eligibility criteria, good baseline comparability and the good quality of the methodological assessment. Further studies are required to more reliably define the role of the tourniquet during TKA, evaluating the impact of the several tourniquet application regimes on clinical and functional outcomes and clarify the association with thromboembolic event.

## Conclusion

For knee arthroplasty longer tourniquet use is associated with shorter surgical duration, lower intra-operative blood lost, higher post-operative haemoglobin values and fewer transfused blood units. The shortest average hospitalisation was associated with no tourniquet use.
