# Correction to: Impact of tourniquet during knee arthroplasty: a bayesian network meta‑analysis of peri‑operative outcomes

**DOI:** 10.1007/s00402-021-03844-w

**Published:** 2021-03-13

**Authors:** Filippo Migliorini, Nicola Maffulli, Paolo Aretini, Andromahi Trivellas, Markus Tingart, Jörg Eschweiler, Alice Baroncini

**Affiliations:** 1 Department of Orthopaedic Surgery, RWTH Aachen University Clinic, Pauwelsstraße 30, 52074 Aachen, Germany; 2 Department of Medicine, Surgery and Dentistry, University of Salerno, Via S. Allende, 84081 Baronissi, SA Italy; 3 School of Pharmacy and Bioengineering, Keele University School of Medicine, Thornburrow Drive, Stoke on Trent, England; 4 Barts and the London School of Medicine and Dentistry, Centre for Sports and Exercise Medicine, Queen Mary University of London, Mile End Hospital, 275 Bancroft Road, London, E1 4DG England; 5 Fondazione Pisana per la Scienza, Via Ferruccio Giovannini, 13, 56017 San Giuliano Terme, Pisa, Italy; 6 Department of Orthopaedics, David Geffen School of Medicine at UCLA, 10833 Le Conte Ave, Los Angeles, CA 90095 USA

## Correction to: Archives of Orthopaedic and Trauma Surgery 10.1007/s00402-020-03725-8

The original version of this article unfortunately contained a mistake. In Fig. 2, one particular aspect of the quality of the article by (Alexandersson et al.) has been wrongly evaluated (Blinding of outcome assessment), and should be instead ‘+’ instead of ‘?’.

The corrected Fig. 2 is given in the following page.

**Fig. 2:**
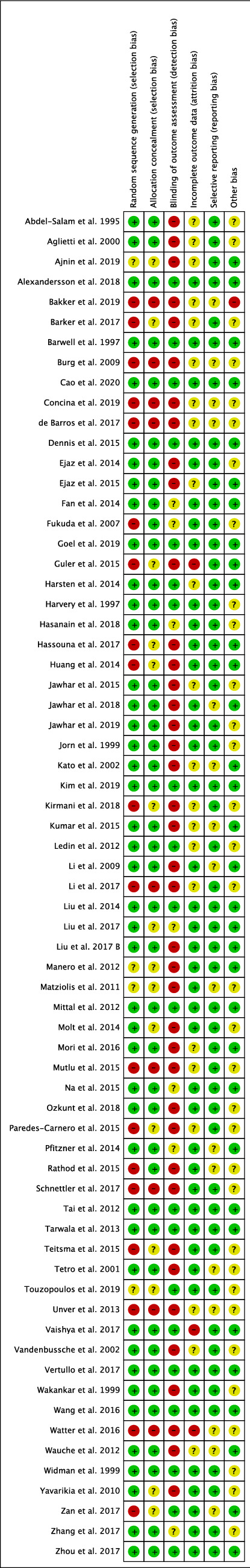
Cochrane risk of bias summary

